# Patient engagement and involvement in rare disease research

**DOI:** 10.1038/s43856-023-00251-7

**Published:** 2023-02-28

## Abstract

Megan O’Boyle is the parent of a 22-year-old daughter with a rare neurodevelopmental disease. She is currently the Patient Engagement Lead of the RARE-X Data Collection Program at Global Genes, a collaborative platform for global data sharing and analysis created to accelerate treatments for rare diseases. In this Q&A, we ask Megan a series of questions on patient engagement and involvement in rare disease research.

**Figure:**
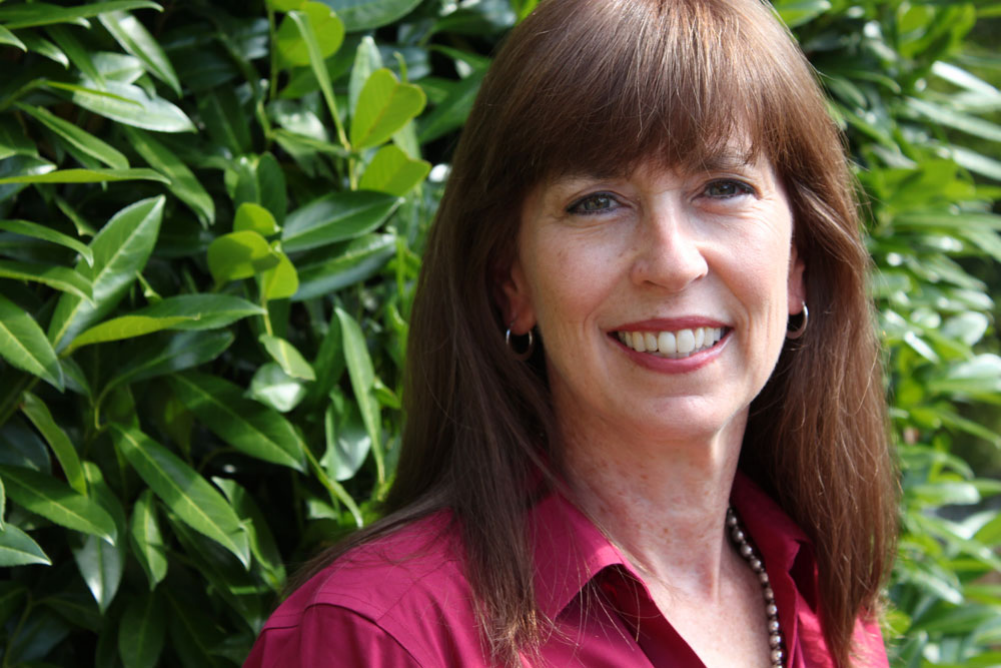
Megan O’Boyle

How have your own personal experiences shaped your commitment to improving patient involvement in rare disease research?

Personally, I am driven to accelerate research across neurodevelopmental disease, including my daughter’s disease, Phelan-McDermid Syndrome, so we can better understand the progression of disease and develop preventions and treatments. Our understanding of many rare diseases and their symptoms is poor. Just when patients and caregivers think they know what to expect, new and often devastating symptoms occur that were not previously described in the literature. For my daughter and many adolescent or adult patients with neurodevelopmental disorders, we were blind-sided by sudden and acute psychiatric regression and catatonia. A disease that was first described three decades ago had not been fully characterized. This meant that no data had been collected and no research was being done.

As the former Principal Investigator for the Phelan-McDermid Syndrome International Registry, I had the opportunity to see first-hand how important high-quality patient-reported data is and how valuable the voice of the patient can be in the drug development process. I also became aware of the issues that can occur when researchers and industry do not involve patients’ perspectives and expertise in the development of protocols, clinical trials schedules, endpoints (i.e. outcomes or measures that allow us to determine whether a treatment has been successful) and other critical aspects of research and drug development.

What are some of the ways in which patients and their caregivers can become involved with research?

The easiest way for most patients and caregivers to begin to get involved in research is through patient advocacy groups or organizations for their disease. As part of these organizations, patient advocates can help to build communities and networks of patients and caregivers, with whom they can share their experiences and get support; develop educational programs for the community or for researchers; and participate in research studies or advise researchers on how to design and carry out their study. The organizations are usually the first place researchers and industry should reach out to before they get too far into their work. These groups have become increasingly savvy and are the trusted partner for the patient community.

What are the benefits of involving patients and their caregivers in research, both for themselves and for researchers?

For researchers and industry, the benefits of involving patients and their caregivers in research are that these individuals are the experts in these diseases. For some rare diseases, there are very few patients and very little existing data, so the experiences and needs of these patients are the primary source of information. Without the insights and feedback of the patient community, researchers may pursue endpoints that are not in fact priorities for the patients and caregivers. Only the patients and their caregivers know first-hand about the range of onset, severity, and debilitation of their symptoms and how these affect their quality of life. Symptoms and severity can vary greatly across patients with the same disease, so it is imperative to get data from as many different patients as possible. Often the patient advocacy groups in the disease space are the best candidates to collect this patient/caregiver-reported data using standardized surveys. The patient community is more likely to respond to the patient advocacy organizations that they are familiar with than to an unknown researcher or industry representative. If there is one central data collection effort with data available to all stakeholders, then the patients will not be as likely to have to contribute the same data over and over again. This is important since survey exhaustion can deter patients from continuing to participate in research.

Patients and caregivers can also benefit from being involved in research in several ways. If patients make their data available, they are more likely to be identified as potential candidates for a clinical trial, which may give them early access to a new treatment. Rare disease communities that contribute patient-reported data to a central data collection on an ongoing basis can also help to contribute to the development of clinical care guidelines. Doctors are often desperate for these, since so little is known about how to care for patients with rare diseases in the clinic. Interacting with researchers in person can also give patients and caregivers the opportunity to share experiences and data with the researchers that they did not ask for but may turn out to be critical information for their research. Finally, for caregivers, participating in research may be a way for them to feel like they are doing something active for their loved one.

How important do you feel it is to collect patient-reported outcomes as part of clinical trials in rare diseases and why?

It is critical to collect patient-reported outcomes as part of clinical trials in rare diseases. Many treatment outcomes are not evident through laboratory tests, imaging, or other biomedical measurements. It is important for the patients to be able to report, preferably in their own words in addition to structured data collection, what their experiences before, during, and after the treatment were. These treatments are not being developed for the benefit of researchers or the pharmaceutical industry, they should be developed for the improved quality of life for the patient. Without patient-reported outcome data, how can the value of the treatment truly be measured?

What are some of the challenges related to patient engagement and involvement in research in this area?

There are many challenges related to patient engagement in rare disease research. Firstly, the number of patients with a given disease is low, so fewer patients are available to engage with research and researchers can have difficulty knowing where to find patients. Patients with some diseases might pass away before being able to participate in research, or there may be a language barrier when trying to engage with patients outside of the researcher’s country. Patient-reported data also needs to be collected through structured or standardized surveys/measures, which is not always the case, to enable researchers to compare data across similar disease types. There is also the issue of funding. Researchers must include funding for patient engagement in grant budgets.

For patients themselves, patient data being held in silos by research or industry organizations will mean that they have to provide the same information to different researchers, creating the potential for survey or research exhaustion. Multiple research efforts occurring simultaneously can confuse or overwhelm patients and caregivers causing them to have decision paralysis, so they choose not to participate in any of the research efforts. Researchers, and sometimes patient advocacy groups, might also use jargon or technical language that can confuse and intimidate patients. Fortunately, many of the above challenges are being addressed by Global Genes’ RARE-X program, and other stakeholders that are committed to lessening the burden to patients, making higher quality data available to researchers and industry, and engaging with the patient community at all stages of the treatment development process.

What do you hope that the future of research on rare diseases looks like?

I hope and believe that, to improve the quality, quantity, and success of rare disease research, with the exception of specific gene editing treatments, research should be done across diseases with similar phenotypes, or characteristics. We will never achieve treatments for the more than 10,000 rare diseases by researching them one disease at a time! Rare diseases are rare, but they are not necessarily unique. Many diseases share clinical characteristics, symptoms, and comorbidities. By grouping these diseases together, patient support, research and the development of treatments could be improved and accelerated.

